# Psychological Toughness of Resistance Fighters: The Palestinian Experience

**DOI:** 10.1192/j.eurpsy.2026.11490

**Published:** 2026-07-31

**Authors:** Y. Zahran, M. Backleh

**Affiliations:** Department of Social and Behavioral Sciences, Birzeit University, Birzeit, West Bank and Gaza

## Abstract

**Introduction:**

Under prolonged occupation, psychological toughness emerges not only as an individual trait but as a collective form of resistance essential for survival and identity. In the Palestinian context, resistance fighters show remarkable resilience despite imprisonment, repression, and trauma. This endurance is cultivated through cultural, social, and political frameworks that transform suffering into strength.

This study explores the socio-political and psychological foundations of such toughness, situated it between lived experience and liberation struggles. By contrasting fighters with non-combatants, it reveals unique coping strategies developed under direct oppression. Using liberation psychology emphasizing dignity, memory, and collective struggle, while also engaging Western resilience theories, the research bridges global psychology with the Palestinian condition.

**Objectives:**

(1) Identify psychological, social, and political factors enhancing toughness; (2) Analyze trauma response differences between fighters and non-combatants; (3) Explore the role of identity and meaning; (4) Propose strategies for civilian resilience-building.

**Methods:**

This study employed a qualitative design integrating liberation psychology with Kobasa’s hardiness model. In-depth interviews with former Palestinian prisoners were thematically analyzed. Based on the findings, a new six-dimension tool (Resistance Psychological Toughness Scale) was developed, which measures: (1) continued resistance, (2) non-submission, (3) breakdown frequency, (4) trauma’s impact on functioning, (5) trauma-induced disorders, and (6) value changes.

**Results:**

Table 1 summarizes the study’s key findings, highlighting core themes, quotations, and their functions from interviews with resistance fighters. The quotations reflect participants’ voices, while the functions show how their words translate into lived realities.

**Table 1.** Core Themes, Quotations, and Functions from Interviews with Resistance Fighters Table 1. Long description.

Theme	Quote	Function
**Meaning**	“Our struggle gives us a reason to endure.”	Turns suffering into purpose stability
**Solidarity**	“We never feel alone.”	Reduces isolation, builds
**Trauma Reframing**	“Every scar reminds us why we resist.”	Converts trauma into motivation
**Spiritual-National Identity**	“Belief in our cause protects us.”	Anchors morale
**Narrative Healing**	“Our stories unite us.”	Collective recovery
**Symbolic Support**	“Without recognition, we feel weaker.”	Maintains resilience

**Conclusions:**

Freedom fighters’ toughness stems from faith, nobility of cause, reframing trauma, transforming pain into collective memory, identity shaped by contrast with non-combatants, and community as moral and practical support.

**Disclosure of Interest:**

None Declared

## Long descriptions

### Table 1. Long description

The table consists of three columns titled Theme, Quote, and Function, with six rows of data.

* Row 1: Theme is Meaning. Quote is ‘Our struggle gives us a reason to endure.’ Function is Turns suffering into purpose stability.

* Row 2: Theme is Solidarity. Quote is ‘We never feel alone.’ Function is Reduces isolation, builds.

* Row 3: Theme is Trauma Reframing. Quote is ‘Every scar reminds us why we resist.’ Function is Converts trauma into motivation.

* Row 4: Theme is Spiritual-National Identity. Quote is ‘Belief in our cause protects us.’ Function is Anchors morale.

* Row 5: Theme is Narrative Healing. Quote is ‘Our stories unite us.’ Function is Collective recovery.

* Row 6: Theme is Symbolic Support. Quote is ‘Without recognition, we feel weaker.’ Function is Maintains resilience.

Navigate back to Table 1..

